# Objective Sleep Quality and the Underlying Functional Neural Correlates Among Older Adults With Probable MCI

**DOI:** 10.1093/geroni/igab046.1461

**Published:** 2021-12-17

**Authors:** Chun Liang Hsu, Ryan Falck, Daniel Backhouse, Patrick Chan, Elizabeth Dao, Lisanne ten Brinke, Brad Manor, Teresa Liu-Ambrose

**Affiliations:** 1 Hinda and Arthur Marcus Institute for Aging Research, Harvard Medical School, Burnaby, British Columbia, Canada; 2 University of British Columbia, Vancouver, British Columbia, Canada; 3 Hinda and Arthur Marcus Institute for Aging Research, Harvard Medical School, Boston, Massachusetts, United States

## Abstract

Poor sleep is a strong risk factor for dementia and is commonly reported among older adults with mild cognitive impairment (MCI). However, the neural underpinnings of poor sleep among older adults with MCI remains equivocal. The goal of this cross-sectional analysis was to explore the relationship between resting-state functional connectivity in the brain and sleep quality as measured by actigraphy. We hypothesize lower sleep efficiency and higher sleep fragmentation may be associated with aberrant functional connectivity of brain regions involved in somatosensory, somatomotor, and attentional processing. Thirty-six community-dwelling older adults with probable MCI between 65-85 years (mean=71.8 years) were assessed for sleep quality using a motion watch to quantify sleep efficiency and fragmentation over 14 days. All participants completed resting-state functional magnetic resonance imaging (fMRI) within 14 days of sleep monitoring. Independent associations between network connectivity and sleep quality were determined using general linear models. Examined networks included the somatosensory network (SMN), dorsal attention network (DAN), ventral attention network (VAN), frontoparietal network (FPN), and default mode network (DMN). Mean Montreal Cognitive Assessment score was 22.5 (SD=2.7) and Mini-Mental State Examination score was 28.3 (SD=1.5). Mean sleep efficiency and fragmentation index was 80.1% and 31.8 respectively. Higher sleep fragmentation correlated with increased connectivity between the SMN and insula, the SMN and posterior cingulate, as well as FPN and primary motor area (Z=3.1; p<0.05). These results suggest aberrant functional connectivity between brain regions involved in attentional and somatosensory processes may be associated with disrupted sleep mechanisms in older adults with MCI.

